# Medical students’ perceptions of the impact of case-based learning on engagement, cognitive skills, and communication

**DOI:** 10.1186/s12909-026-09316-2

**Published:** 2026-04-28

**Authors:** Fahad Abdulaziz Alrashed, Tauseef Ahmad, Saad M. Alangari, Mohammed A. Albabtain, Abdullah S. Aldughaither, Rashed Alrashed, Ahmed Othman Alsabih, Hamza M. Abdulghani

**Affiliations:** 1 Department of Medical Education, College of Medicine, King Saud University, Imperial College London, P.O Box 2925, Riyadh, Riyadh 11461 Saudi Arabia; 2 College of Medicine, King Saud University, P.O.Box 2925, Riyadh, Riyadh 11461 Saudi Arabia; 3 Department of Internal medicine, College of Medicine, King Saud University, P.O.Box 2925, Riyadh, Riyadh 11461 Saudi Arabia; 4 Department of Physiology, College of Medicine, King Saud University, P.O. Box 2925, Riyadh, Riyadh 11461 Saudi Arabia; 5 Department of Medical Education and Family Medicine, College of Medicine, King Saud University, P.O.Box 2925, Riyadh, Riyadh, Riyadh 11461 Saudi Arabia

**Keywords:** Case-based Learning, Medical Education, Health-Care Education, Critical Thinking, PBL, FDP, Perceived benefits of CBL

## Abstract

**Background:**

Medical education has shifted from traditional lectures to student-centered approaches, with case-based learning (CBL) gaining prominence. CBL engages students in real-world clinical scenarios, fostering critical thinking, problem-solving, and clinical reasoning. Previously published studies show CBL enhances knowledge retention and improves decision-making skills compared to traditional methods. In Saudi Arabia, CBL is increasingly adopted in alignment with Vision 2030 reforms. However, challenges such as resource limitations and engagement strategies remain. Despite its benefits, the implementation of CBL within the Saudi context is underexplored. This manuscript is to evaluate the effectiveness of CBL in enhancing medical students’ perceived benefits of CBL, with a focus on their engagement levels and the perceived impact on problem-solving, critical thinking, clinical reasoning, and communication skills, as well as to identify the challenges faced in adapting to the CBL format.

**Method:**

A descriptive cross-sectional study was conducted from January to April 2025 among third- to fifth-year clinical medical students at King Saud University, Riyadh. A self-administered questionnaire with 19 items was developed based on a literature review and expert input. It was distributed online via Google Forms to eligible students through email and WhatsApp. The questionnaire covered demographics, engagement with case-based learning (CBL), perceptions of CBL, and challenges faced. Data were analyzed using SPSS version 24, applying descriptive statistics and correlation analysis, with significance set at *p* < 0.05.

**Results:**

A total of 327 clinical-year medical students participated, with 68.5% reporting that case-based learning (CBL) improved critical thinking. Engagement varied, but higher engagement was significantly associated with greater satisfaction, better understanding, and enhanced skills (*p* < 0.001). Most students believed CBL improved problem-solving (59.3%), knowledge retention (60.9%), and teamwork (53.2%). However, challenges included difficulty accessing resources (30.3%) and adapting to the CBL format. Significant correlations were observed among critical thinking, deep learning, and bridging theoretical-practical gaps (*r* = 0.78), highlighting CBL’s positive educational impact.

**Conclusion:**

CBL positively influenced medical students’ engagement, understanding, and development of essential clinical skills, including clinical reasoning, decision-making, teamwork, and communication skills. These skills are crucial for effective medical practice and which is getting enhanced through active participation in CBL sessions. Higher engagement levels were significantly associated with improved satisfaction and perceived benefits of CBL. Despite these benefits, challenges such as resource limitations and adaptation difficulties were reported. These findings highlight the need for structured implementation, faculty training, and improved support systems to optimize the effectiveness of CBL in medical education.

**Supplementary Information:**

The online version contains supplementary material available at 10.1186/s12909-026-09316-2.

## Introduction

Medical education has evolved significantly from traditional lecture-based methods to more interactive and student-centered approaches. Case-based learning (CBL) has emerged as a prominent pedagogical strategy, emphasizing real-world clinical scenarios to enhance critical thinking, problem-solving, and clinical reasoning skills among medical students. CBL is an instructional method that uses real-world cases to promote active learning in medical education. Over recent decades, CBL has emerged as a valuable pedagogical tool in medical schools, providing a bridge between theoretical knowledge and clinical practice. CBL is designed to enhance the development of various cognitive skills, including critical thinking, problem-solving, clinical reasoning, and retention of medical knowledge [1–3]. One of the primary benefits of CBL is its ability to promote critical thinking [4].

CBL encourages students to evaluate and analyze real-life scenarios, thus enhancing their ability to think critically [5]. When students work through complex clinical cases, they must identify key issues, formulate hypotheses, and make decisions based on available evidence, all of which are fundamental aspects of critical thinking [4, 6]. A study showed that students exposed to case-based teaching performed better in problem-solving tasks compared to those who underwent traditional lectures [7]. This finding underscores the importance of CBL in providing students with the opportunity to solve real-world problems, which directly translates to improved clinical decision-making. Recent studies have shown that students exposed to these innovative forms of case-based teaching outperform their peers who rely on traditional lecture-based learning methods, particularly in problem-solving and clinical reasoning skill [4, 5]. This approach has been shown to foster a deeper understanding of medical concepts by facilitating the active application of knowledge, thus making learning more meaningful and effective [8, 9]. Retention of medical knowledge is also a critical concern in medical education, as the vast amount of information students must learn can easily be forgotten over time. CBL offers a solution by promoting active learning and repetition of key concepts within the context of clinical cases. Students who participated in CBL had higher retention rates of medical knowledge compared to those who learned through traditional methods, such as lectures and textbooks [10, 11]. The interactive nature of CBL, combined with its focus on practical application, helps reinforce students’ memory of essential medical information [12].

A theoretical perspective, the relationship between student engagement and perceived benefits of CBL in case-based learning (CBL) can be explained through constructivist learning theory, which posits that knowledge is actively constructed through meaningful interaction with learning materials rather than passively received [13]. In CBL, students engage with authentic clinical cases that require analysis, hypothesis generation, and decision-making, thereby promoting deeper cognitive processing and the development of higher-order thinking skills such as critical thinking and clinical reasoning [14, 15]. The social learning theory emphasizes the role of interaction and collaboration in learning [16]. CBL typically involves group discussions and peer collaboration, in which higher levels of engagement facilitate shared problem-solving, communication, and reflection, leading to enhanced perceptions of communication and collaborative skills [17]. Student engagement theory further supports this relationship by suggesting that students who are behaviorally, cognitively, and emotionally engaged are more likely to invest effort, persist in learning tasks, and perceive greater educational benefits [18, 19].

Saudi Arabia’s medical education system is currently undergoing significant transformation toward student-centered learning, in line with Vision 2030’s broader reforms in higher education and healthcare pedagogy [20]. A study comparing CBL and lecture-based learning in pharmacology instruction at King Saud Bin Abdulaziz University for Health Sciences found that CBL improved student engagement and perceived benefits of CBL [12]. King Saud University’s medical curriculum has undergone significant transformation in recent years, with a strong emphasis on student-centered learning, in alignment with the Vision 2030 reforms in Saudi Arabia. Case-Based Learning (CBL) plays a pivotal role in this transformation, by encouraging students to actively engage in clinical reasoning, problem-solving, and decision-making. This study investigates the implementation of CBL in this unique context, with an emphasis on how the curriculum design and teaching strategies are tailored to enhance students’ perceived benefits of CBL in line with the national education vision. Despite the well-documented benefits of CBL, its implementation in the Saudi context presents unique challenges, including limited resources and technological constraints. The alignment of CBL with the broader Vision 2030 educational reforms aims to bridge these gaps and provide an enhanced learning experience for medical students, preparing them for the evolving demands of healthcare in Saudi Arabia.

While existing studies highlight the benefits of CBL in medical education, there is a paucity of research focusing on the specific challenges encountered in its implementation within Saudi Arabia. Issues such as resource limitations, technological barriers, and strategies to maintain student engagement require further investigation. Additionally, the role of CBL in enhancing clinical reasoning and bridging the gap between theoretical knowledge and practical application in the Saudi medical curriculum remains underexplored. Addressing these gaps is crucial for optimizing CBL’s effectiveness and ensuring its successful integration into medical education programs in Saudi Arabia. The objective of this study is to evaluate the effectiveness of CBL in enhancing medical students’ perceived benefits of CBL, with a focus on their engagement levels and the perceived impact on problem-solving, critical thinking, clinical reasoning, and communication skills, as well as to identify the challenges faced in adapting to the CBL format.

## Methods

This descriptive cross-sectional study was conducted from January to April 2025 among third- to fifth-year clinical medical students at King Saud University, Riyadh. Participants were selected using a purposive sampling technique. This sampling method was chosen to ensure that the study included students with direct experience in the CBL format within the clinical curriculum. The study population consisted of all third- to fifth-year medical students during the 2024/2025 academic year. Students from other faculties or institutions, those who did not complete the survey, first- and second-year (pre-clinical) students, medical interns (graduates), and individuals enrolled in the university’s preparatory year were excluded. A minimum sample size of 287 students was determined based on prior studies, ensuring adequate statistical power. For the purposes of this study, ‘engagement’ was defined not only by attendance at CBL sessions but also by students’ self-reported active involvement in various components of CBL, such as preparation for the cases, participation in group discussions, and involvement in collaborative problem-solving. To assess engagement, students were asked to report how often they participated in these activities using a five-point scale. By using this self-reported scale, we were able to categorize students based on their perception of their own active participation, rather than simply their attendance. This approach allowed us to capture a more nuanced view of engagement that goes beyond mandatory attendance and reflects the depth of students’ involvement in the CBL format. The sample size estimation was derived from a few previous studies [12, 21, 22]. Applying the formula based on the average proportion response rate (0.76) with a 95% confidence level and a 5% margin of error, the minimum sample size was calculated to be 242. To account for potential non-responses or incomplete questionnaires, an additional 20% was added, increasing the required sample to 287.

### Study instrument

The study used a self-administered questionnaire developed based on a literature review and expert input. A panel of medical education professors and CBL experts refined the instrument, resulting in a final set of nineteen items. The study participants were not directly involved in the initial stages of instrument development. However, their feedback was incorporated during the pilot testing phase. A pilot test was conducted with 19 participants to evaluate the instrument’s clarity and reliability (a Cronbach’s alpha coefficient of 0.836, indicating strong internal consistency). Following this feedback, modifications were implemented to improve the questionnaire’s effectiveness, resulting in a final version with nineteen items. This process helped ensure that the questionnaire was both relevant and user-friendly for the target population. The study questionnaire was distributed to all clinical-year medical students (third to fifth year). An English version of the questionnaire, created in Google Forms, was shared with students via email and WhatsApp groups. The first section of the questionnaire included four questions related to demographic information and engagement in CBL. For the purposes of this study, “engagement” was defined as the frequency with which students actively participated in CBL sessions, including attending sessions, preparing for cases in advance, contributing to group discussions, and participating in collaborative problem-solving activities. Participants were asked to report how often they engaged in CBL on a five-point scale: very frequently, frequently, occasionally, and rarely. This definition was clearly provided in the instructions preceding the engagement questions to ensure consistency in understanding among participants. The second section consisted of eleven items assessing students’ perceptions of CBL, measured using a five-point Likert scale with response options: strongly agree, agree, neutral, disagree, and strongly disagree. The final section focused on the challenges encountered during the implementation of CBL. Informed consent, clearly outlining the study’s purpose, was obtained from all participants at the start of the questionnaire. Participants were assured that all data would remain anonymous and confidential, with each respondent assigned a unique code solely for analysis purposes. No incentives or rewards were provided for participation (Appendix 1).

### Study variables

The questionnaire included demographic data (gender, academic year, and CBL engagement frequency) and assessed perceptions of CBL (satisfaction, skill development, and challenges). The dependent variable was the frequency of CBL engagement, and the study examined its relationship with independent variables such as perceptions of skill development and challenges encountered during CBL implementation. Based on participants’ responses, we examined the relationship between engagement frequency and the independent variables, as well as the distribution of responses across these variables. Additionally, we identified the challenges faced by students in adapting to CBL and compared these challenges between students who frequently engaged with CBL and those who did not. Based on prior previous literature using similar parameters, the required sample size for this study was estimated to be approximately 280–300 participants.

### Educational context of case-based learning

At King Saud University’s College of Medicine, CBL is a mandatory component of the clinical-phase curriculum (third to fifth years). CBL sessions are integrated into selected clinical courses, which are designed to complement traditional lectures and clinical rotations. The frequency of CBL sessions varies depending on the course, with most clinical courses incorporating CBL sessions on a bi-weekly basis. During these sessions, small groups of students are presented with clinical cases that align with the course’s learning objectives. Students are required to prepare in advance by reviewing assigned readings and clinical cases. The sessions are facilitated by faculty members, who guide the group discussions, encourage critical thinking, and assist students in applying theoretical knowledge to clinical practice.

### Ethics approval and consent to participate

This study was approved by the Institutional Review Board (IRB) at King Saud University (KSU) in Riyadh, Saudi Arabia (Ethics Approval No. KSU-HE-24-390). Before participating, all participants signed a consent form. All participants provided informed consent prior to data collection. Participation was voluntary. The methods used were in accordance with all relevant guidelines and regulations. The study was conducted in accordance with the principles of the Declaration of Helsinki and all relevant guidelines and regulations.

### Data analysis

The collected data were analyzed using IBM SPSS Statistics for Windows, version 24.0. Descriptive statistics, including frequencies and percentages, were employed to summarize participants’ socio-demographic characteristics in tabular form. Categorical variables such as engagement with CBL, satisfaction level, perceived enhancement of medical concepts through CBL, and challenges in CBL implementation were also summarized and analyzed. That chi-squared tests were used to examine associations between engagement frequency and categorical outcome variables, and that all engagement levels were retained as separate categories in the analysis. Relationships between these variables were examined using correlation analysis, with statistical significance set at a p-value of 0.05.

## Results

### Participant characteristics and response rate

A total of 478 medical students were invited to participate in the study, of whom 351 responded, yielding a response rate of 68.4%. Data from 24 respondents were excluded due to missing information. The final analysis included 327 medical students. Most participants were male (65.7%), and the largest portion of participants were 5th-year students (45.3%), followed by 3rd-year students (30.3%) and 4th-year students (24.5%), as shown in Table 1.

**Table 1:** Students’ perception about the CBL

Items	categories	*n*(%)
Gender	Male	215(65.7)
	Female	112(34.3)
Academic year	3rd year	99(30.3)
	4th year	80(24.5)
	5th year	148(45.3)
How often have you been engaged in case-based learning in your medical curriculum?	Rarely	20(6.1)
	Occasionally	83(25.4)
	Frequently	156(47.7)
	Very Frequently	68(20.8)
I am satisfied with the case-based learning sessions.	Agree	132(40.4)
	Neutral	119(36.4)
	Disagree	76(23.2)
I believe that case-based learning enhances my understanding of medical concepts.	Agree	219(67.0)
	Neutral	56(17.1)
	Disagree	52(15.9)
Using case-based learning is beneficial for deep learning and developing critical thinking skills.	Agree	224(68.5)
	Neutral	49(15.0)
	Disagree	54(16.5)
The CBL session provides the platform for developing and understanding the sound knowledge of a core subject.	Agree	193(59.0)
	Neutral	78(23.9)
	Disagree	56(17.1)
I believe that the skills acquired from case-based learning sessions would help me prepare for a future career in medicine.	Agree	189(57.8)
	Neutral	74(22.6)
	Disagree	64(19.6)
Case-based learning sessions improved my problem-solving skills.	Agree	194(59.3)
	Neutral	83(25.4)
	Disagree	50(15.3)
Case-based learning has contributed to better retention of medical knowledge compared to traditional teaching methods.	Agree	199(60.9)
	Neutral	71(21.7)
	Disagree	57(17.4)
The discussion during the CBL sessions improved my teamwork skills.	Agree	174(53.2)
	Neutral	79(24.2)
	Disagree	74(22.6)
I believe my clinical reasoning has improved with CBL sessions.	Agree	200(61.2)
	Neutral	83(25.4)
	Disagree	44(13.5)
The adoption of technology (e.g., virtual cases, and simulations) enhances the effectiveness of case-based learning in clinical teaching and clerkship training.	Agree	214(65.4)
	Neutral	67(20.5)
	Disagree	46(14.1)
I feel that case-based learning helps bridge the gap between theoretical knowledge and its practical application in real-world clinical scenarios.	Agree	228(69.7)
	Neutral	62(19.0)
	Disagree	37(11.3)

### Engagement with case-based learning

Levels of engagement with case-based learning varied among participants. Nearly half of the students (47.7%) reported frequent engagement with CBL, while 20.8% reported very frequent engagement. Overall perceptions of CBL were positive, with 67.0% of participants indicating that CBL enhanced their understanding of medical concepts.

### perceived impact of cbl on cognitive and professional skills

Students reported substantial perceived benefits of CBL in developing key cognitive and professional skills. Improvements were reported in critical thinking (68.5%), bridging theoretical knowledge with clinical practice (69.7%), retention of medical knowledge (60.9%), problem-solving skills (59.3%), and teamwork abilities (53.2%). Additionally, 65.4% of students agreed that the integration of technology, such as virtual cases and simulations, enhanced the effectiveness of CBL for clinical training (Table 1).

### Perceived benefits of and challenges associated with CBL

Table 2 Summarizes students’ perceptions of the benefits of CBL and its associated challenges. Regarding collaboration with peers, 40.7% of students reported moderate encouragement, while 23.9% stated that CBL activities encouraged collaboration to a great extent. A smaller proportion, 13.1%, found that CBL significantly promoted peer collaboration, while 6.7% reported no encouragement at all. When asked about the development of communication skills in a medical context, 36.7% of students noted moderate improvement, and 29.1% indicated significant improvement. However, 19.0% reported slight improvement, while 4.3% found no impact on their communication skills. Challenges in understanding and adapting to the CBL format were common. A significant portion of students (25.4%) struggled with synthesizing information, while 24.8% expressed uncertainty about the relevance of the cases to clinical practice. Additionally, 29.1% cited other factors that made adapting to the format challenging. Accessing necessary resources for CBL was also an issue for many students, with 30.3% experiencing limited access to relevant materials, and 33.6% facing other resource-related problems. The lack of adequate technological support (21.7%) and library resources (14.4%) were additional barriers.

**Table 2:** Students’ perceptions of the benefits and challenges associated with case-based learning (CBL)

Items	Categories	*n*(%)
To what extent do case-based learning activities encourage collaboration with your peers?	Not at All	22(6.7)
	Slightly	51(15.6)
	Moderately	133(40.7)
	Very much	78(23.9)
	Extremely	43(13.1)
How has case-based learning contributed to the development of your communication skills in a medical context?	Not at All	14(4.3)
	Slightly	62(19.0)
	Moderately	120(36.7)
	Very much	95(29.1)
	Extremely	36(11.0)
In your experience, what challenges, if any, have you encountered in understanding and adapting to the case-based learning format?	Lack of clarity in case presentation	68(20.8)
	Difficulty in synthesizing information	83(25.4)
	Uncertainty about the relevance of cases to clinical practice	81(24.8)
	Others factors	95(29.1)
Were there any difficulties in accessing or utilizing the necessary resources for case-based learning?	Limited access to relevant materials	99(30.3)
	Insufficient technological support	71(21.7)
	Lack of adequate library resources	47(14.4)
	Other resource problem	110(33.6)
Have there been challenges in maintaining student engagement and motivation throughout case-based learning sessions?	Challenges in connecting theoretical concepts to practical applications	78(23.9)
	Difficulty in sustaining interest	190(58.1)
	Others challenges	59(18.0)

### Association between engagement and perceived benefits of CBL

Table 3 summarizes the participants’ perceptions of CBL regarding their engagement with the CBL curriculum. Higher engagement levels were linked to greater satisfaction. Among those frequently engaged, 45.6% were satisfied, and 55.9% of those who were very frequently engaged expressed satisfaction. The Chi-square test (P-value < 0.001) highlighted a significant relationship between engagement and satisfaction. CBL was perceived as enhancing understanding, with 73.1% of frequently engaged and 75.0% of very frequently engaged students agreeing. Chi-square results (P-value < 0.001) confirmed that greater engagement is significantly correlated with better understanding of medical concepts. Most students with higher engagement (77.0% for frequently and 72.1% for very frequently) agreed that CBL developed their critical thinking skills. This association was also statistically significant (P-value < 0.001). Higher engagement was positively associated with improvements in problem-solving (62.2% for frequently and 76.5% for very frequently engaged students) and better retention of knowledge (66.7% for frequently and 66.2% for very frequently engaged). Both relationships were significant (P-value < 0.001). Furthermore, higher engagement was positively associated with improvements in problem-solving (62.2% for frequently and 76.5% for very frequently engaged students) and better retention of knowledge (66.7% for frequently and 66.2% for very frequently engaged). Both relationships were significant (P-value < 0.001). Moreover, the majority of highly engaged students (63.5% for frequently and 48.6% for very frequently engaged) felt CBL improved teamwork and clinical reasoning skills. Chi-square tests confirmed significant associations (P-value < 0.001).

**Table 3:** The participants’ perceptions about case-based learning (CBL) and their level of engagement with the CBL curriculum

Items	Categories	How often have you been engaged in case-based learning in your medical curriculum?				Chi-squire
		Rarely	Occasionally	Frequently	Very Frequently	*P*-value
I am satisfied with the case-based learning sessions.	Agree	0(0.0)	23(27.8)	71(45.6)	38(55.9)	40.4(< 0.001)
	Neutral	8(40.0)	32(38.6)	62(39.8)	17(25.0)	
	Disagree	12(60.0)	28(33.8)	23(14.8)	13(19.2)	
I believe that case-based learning enhances my understanding of medical concepts.	Agree	6(30.0)	48(57.9)	114(73.1)	51(75.0)	45.2(< 0.001)
	Neutral	2(10.0)	25(30.2)	23(14.8)	6(8.9)	
	Disagree	12(60.0)	48(57.9)	19(12.2)	11(16.2)	
Using case-based learning is beneficial for deep learning and developing critical thinking skills.	Agree	4(20.0)	51(61.5)	120(77.0)	49(72.1)	40.1(< 0.001)
	Neutral	4(20.0)	18(21.7)	20(12.9)	7(10.3)	
	Disagree	12(60.0)	14(16.9)	16(10.3)	12(17.7)	
The CBL session provides the platform for developing and understanding the sound knowledge of a core subject.	Agree	2(10.0)	47(56.6)	100(64.1)	44(64.7)	46.1(< 0.001)
	Neutral	4(20.0)	24(29.0)	37(23.8)	13(19.2)	
	Disagree	14(70.0)	12(14.5)	19(12.2)	11(16.2)	
I believe that the skills acquired from case-based learning sessions would help me prepare for a future career in medicine.	Agree	8(40.0)	36(43.4)	108(69.3)	37(54.5)	26.1(< 0.001)
	Neutral	4(20.0)	28(33.8)	30(19.3)	12(17.7)	
	Disagree	8(40.0)	19(22.9)	18(11.6)	19(28.0)	
Case-based learning sessions improved my problem-solving skills.	Agree	4(20.0)	41(49.4)	97(62.2)	52(76.5)	50.0(< 0.001)
	Neutral	4(20.0)	32(38.6)	40(25.7)	7(10.3)	
	Disagree	12(60.0)	10(12.1)	19(12.2)	9(13.3)	
Case-based learning has contributed to better retention of medical knowledge compared to traditional teaching methods.	Agree	6(30.0)	44(53.1)	104(66.7)	45(66.2)	31.7(< 0.001)
	Neutral	2(10.0)	24(29.0)	32(20.6)	13(19.2)	
	Disagree	12(60.0)	15(18.1)	20(12.9)	10(14.7)	
The discussion during the CBL sessions improved my teamwork skills.	Agree	2(10.0)	40(48.2)	99(63.5)	33(48.6)	35.2(< 0.001)
	Neutral	6(30.0)	17(20.5)	32(20.6)	24(35.3)	
	Disagree	12(60.0)	26(31.4)	25(16.1)	11(16.2)	
I believe my clinical reasoning has improved with CBL sessions.	Agree	6(30.0)	47(56.7)	105(67.3)	42(61.8)	29.0(< 0.001)
	Neutral	4(20.0)	22(26.5)	38(24.4)	19(28.0)	
	Disagree	10(50.0)	14(16.9)	13(8.4)	7(10.3)	
The adoption of technology (e.g., virtual cases, and simulations) enhances the effectiveness of case-based learning in clinical teaching and clerkship training.	Agree	6(30.0)	46(55.5)	112(71.8)	50(73.6)	36.4(< 0.001)
	Neutral	6(30.0)	20(24.1)	36(23.1)	5(7.4)	
	Disagree	8(40.0)	17(20.5)	8(5.2)	13(19.2)	
I feel that case-based learning helps bridge the gap between theoretical knowledge and its practical application in real-world clinical scenarios.	Agree	4(20.0)	53(63.9)	124(79.5)	47(69.2)	67.5(< 0.001)
	Neutral	4(20.0)	21(25.3)	28(18.0)	9(13.3)	
	Disagree	12(60.0)	9(10.9)	4(2.6)	12(17.7)	

### Engagement and perceived challenges

Table 4 reports participants’ perceptions of CBL challenges about their engagement with the CBL curriculum. As the engagement with CBL increased, so did the perception of collaboration. Students who engaged frequently (47.4%) or very often (30.9%) reported moderate to very much collaboration, whereas those with lower engagement (rarely and occasionally) reported lower levels of cooperation. This trend was consistent across all categories. Furthermore, higher engagement was positively correlated with improved communication skills. Among students with frequent (42.9%) and persistent (32.4%) engagement, many agreed that CBL enhanced their communication in a medical context. Moreover, difficulty in accessing relevant materials increased with lower engagement levels. Students who engaged frequently (28.8%) or very often (36.8%) reported more challenges with resource access than those who engaged less regularly.

**Table 4:** The participants’ perceptions about case-based learning (CBL) challenges, regarding their level of engagement with the CBL curriculum

Factor: To what extent do case-based learning activities encourage collaboration with your peers?						
Items	**Categories**	**Not at all**	**Slightly**	**Moderately**	**Very much**	**Extremely**
How often have you been engaged in case-based learning in your medical curriculum?	Rarely	6(30.0)	8(40.0)	2(10.0)	4(20.0)	0(0.0)
	Occasionally	8(9.6)	18(21.7)	36(43.4)	15(18.1)	6(7.2)
	Frequently	8(5.1)	15(9.6)	74(47.4)	41(26.3)	18(11.5)
	Very Frequently	0(0.0)	10(14.7)	21(30.9)	18(26.5)	19(27.9)

**Factor: How has case-based learning contributed to the development of your communication skills in a medical context?**						
	**Categories**	**Not at all**	**Slightly**	**Moderately**	**Very much**	**Extremely**
How often have you been engaged in case-based learning in your medical curriculum?	Rarely	6(30.0)	8(40.0)	2(10.0)	4(20.0)	0(0.0)
	Occasionally	1(1.2)	27(32.5)	37(44.6)	16(19.3)	2(2.4)
	Frequently	1(0.6)	21(13.5)	67(42.9)	53(34.0)	14(9.0)
	Very Frequently	6(8.8)	6(8.8)	14(20.6)	22(32.4)	20(29.4)

**Factor: In your experience**,** what challenges**,** if any**,** have you encountered in understanding and adapting to the case-based learning format?**						
	**Categories**	**Lack of clarity in case presentation**	**Difficulty in synthesizing information**	**Uncertainty about the relevance of cases to clinical practice**	**Others**	
How often have you been engaged in case-based learning in your medical curriculum?	Rarely	4(20.0)	4(20.0)	6(30.0)	6(30.0)	
	Occasionally	12(14.5)	19(22.9)	15(18.1)	37(44.6)	
	Frequently	34(21.8)	48(30.8)	36(23.1)	38(24.4)	
	Very Frequently	18(26.5)	12(17.6)	24(35.3)	14(20.6)	

**Factor: Were there any difficulties in accessing or utilizing the necessary resources for case-based learning?**						
	**Categories**	**Limited access to relevant materials**	**Insufficient technological support**	**Lack of adequate library resources**	**Others**	
How often have you been engaged in case-based learning in your medical curriculum?	Rarely	2(10.0)	4(20.0)	4(20.0)	10(50.0)	
	Occasionally	29(34.9)	10(12.0)	13(15.7)	31(37.3)	
	Frequently	45(28.8)	43(27.6)	24(15.4)	44(28.2)	
	Very Frequently	21(36.8)	14(20.6)	8(11.8)	25(36.8)	

**Factor: Have there been challenges in maintaining student engagement and motivation throughout case-based learning sessions?**						
Items	**Categories**	**Difficulty in sustaining interest**	**Challenges in connecting theoretical concepts to practical applications**	**Others**		
How often have you been engaged in case-based learning in your medical curriculum?	Rarely	12(60.0)	2(10.0)	6(30.0)		
	Occasionally	15(66.3)	18(21.7)	10(12.0)		
	Frequently	89(57.1)	37(23.7)	30(19.2)		
	Very Frequently	34(50.0)	21(30.9)	13(19.1)		

### Correlation analysis of CBL factors

Supplementary table shows the correlation coefficients between various factors of CBL, highlighting the relationships among students’ perceptions of CBL and its effectiveness. All factors exhibit significant positive correlations (P-value < 0.001), indicating strong associations between the perceived benefits of CBL and student engagement. The highest correlation was observed between “Using case-based learning for deep learning and developing critical thinking skills” and “Case-based learning helps bridge the gap between theoretical knowledge and its practical clinical scenarios” (r = 0.78), suggesting that CBL is highly effective in promoting both critical thinking and knowledge retention. Similarly, “Case-based learning enhances understanding of medical concepts” was strongly correlated with “Case-based learning improves problem-solving skills” (r = 0.64), emphasizing the interconnectedness of CBL’s impact on different perceived benefits of CBL. Other significant correlations include “I am satisfied with CBL sessions” and “I believe my clinical reasoning has improved with CBL” (r = 0.49), indicating that overall satisfaction with CBL is linked to perceived improvements in clinical reasoning. Additionally, “The adoption of technology enhances the effectiveness of CBL” showed a moderate correlation with I believe CBL helps bridge the gap between theoretical knowledge and its practical application” (r = 0.39), suggesting that integrating technology into CBL positively affects its practical application.

## Discussion

### Engagement with case-based learning

This study examined medical students’ engagement with case-based learning (CBL) and its perceived impact on learning and skill development. The findings demonstrate considerable variability in student engagement, with nearly half of participants reporting frequent engagement and approximately one-fifth reporting very frequent engagement. Such variation has been observed in previous studies and may be influenced by differences in course design, faculty facilitation styles, workload distribution, and students’ individual learning strategies [23, 24]. Despite this variability, overall engagement with CBL was generally high, supporting its acceptability within the medical curriculum.

### Perceived educational benefits of CBL

Overall, students reported favorable perceptions of CBL, particularly regarding understanding medical concepts. A substantial proportion perceived CBL as beneficial for enhancing comprehension, problem-solving, knowledge retention, teamwork, and critical thinking. These findings align with prior research demonstrating that CBL promotes active learning and supports the development of higher-order cognitive skills compared with traditional lecture-based approaches [25, 26]. The strong perception that CBL bridges theoretical knowledge with practical clinical application further underscores its relevance to clinical training. This is consistent with evidence suggesting that contextualized learning through authentic cases enhances clinical reasoning and prepares students for real-world decision-making [27, 28]. The reported value of technology-enhanced CBL, including virtual cases and simulations, also mirrors findings in the literature highlighting the role of digital tools in providing safe, interactive environments that foster deeper engagement and repeated practice [29, 30].

### Challenges associated with CBL implementation

Despite the overall positive perceptions, the study identified several challenges related to CBL implementation. Students reported varying levels of peer collaboration, reflecting the influence of group dynamics and facilitator effectiveness on perceived benefits of CBL. Previous studies similarly emphasize that dominant group members, uneven participation, or insufficient facilitation can hinder meaningful engagement during CBL sessions [31].

Difficulties in synthesizing information and uncertainty regarding the clinical relevance of some cases were also reported. These challenges are commonly observed during transitions to student-centered instructional methods and may reflect cognitive overload or insufficient scaffolding during early exposure to CBL [32, 33]. Additionally, limitations in accessing learning resources and technological support were noted, suggesting that institutional infrastructure plays a critical role in maximizing the effectiveness of CBL. Furthermore, through CBL communication skills enhancement, there was a moderate to significant improvement in the current study. These findings align with a quasi-experimental study in nursing education illustrating that CBL significantly enhances both communication skills and problem-solving abilities compared to lecture-based approaches [34].

### Relationship between engagement and perceived benefits of CBL

The current study’s findings were the strong positive association between students’ level of engagement with CBL and their satisfaction, perceived knowledge gains, and skill development. Students who engaged more frequently with CBL were significantly more likely to report enhanced understanding, improved critical thinking, problem-solving abilities, teamwork, and clinical reasoning skills. These findings are consistent with previous research demonstrating that active participation in CBL fosters deeper learning and greater perceived relevance of course content [1, 27, 28, 35]. The observed associations between engagement and knowledge retention further support the premise that active learning environments promote long-term learning through repeated application and contextual reinforcement of concepts [9, 36, 37]. It was found that the interactive and collaborative nature of CBL fosters analytical thinking and diagnostic reasoning abilities essential for clinical practice. The significant correlation between engagement and teamwork skills observed in this study also aligns with findings suggesting that group-based problem-solving fosters communication and collaborative competencies [34]. The positive link between CBL engagement and improved retention of knowledge aligns with previous studies [34, 38], which emphasize that active learning environments enhance memory consolidation through repeated application of concepts across varied contexts. Similarly, a strong correlation (*r* = 0.64) between “CBL enhances understanding of medical concepts” and “CBL improves problem-solving skills” underscores the interrelation of cognitive outcomes. Active learning strategies such as like CBL are well-documented for facilitating deeper comprehension and effective problem-solving [39, 40]. Furthermore, satisfaction with CBL sessions and improvements in clinical reasoning (*r* = 0.49) suggest that learner satisfaction supports perceived gains in reasoning abilities. Studies report that when learners feel supported and engaged through CBL, they are more likely to develop sound clinical judgment [41, 42].

### Educational implications

The significant associations between engagement, satisfaction, and perceived benefits of CBL highlight the importance of fostering active participation in CBL sessions. Well-designed cases, trained facilitators, manageable group sizes, and adequate technological and learning resources may help optimize student engagement and enhance perceived benefits of CBL. These findings reinforce the need for faculty development and institutional support to ensure the effective implementation of CBL within medical curricula.

### Limitations

This study has several limitations. First, it relied on self-reported data, which may be subject to response bias. Second, the cross-sectional design limits the ability to establish causal relationships between CBL engagement and skill development. Additionally, the study was conducted at a single institution, which may affect the generalizability of the findings to other medical schools or educational contexts. Another limitation is the potential for selection bias. Students who are already interested in the CBL format or aware of its benefits may be more likely to engage in CBL activities, which could influence the results. This selection bias may limit the sample’s representativeness and the ability to generalize the findings to all students.

## Conclusion and recommendations

This study demonstrates that case-based learning (CBL) is an effective student-centered approach that enhances medical students’ understanding of clinical concepts, critical thinking, problem-solving, clinical reasoning skill, and communication skills. Higher levels of engagement with CBL were strongly associated with greater satisfaction and improved perceived educational outcomes, highlighting the importance of active participation for maximizing learning benefits. Despite these positive effects, students reported challenges, including difficulties accessing learning resources, adapting to the CBL format, and variability in peer collaboration. These findings underscore the need for structured and supportive implementation strategies.

To maximize the benefits of CBL, medical schools should implement faculty development programs to ensure consistent and skilled facilitation, integrate CBL systematically throughout the curriculum to reinforce learning objectives, and provide students with orientation sessions on collaborative learning and expectations for active engagement. Enhancing access to technological tools, virtual cases, and library resources can support case preparation and participation, while maintaining manageable group sizes can foster effective peer collaboration. Additionally, establishing regular feedback and evaluation mechanisms will help monitor student experiences, identify challenges, and continuously refine CBL activities, ensuring that students derive the maximum educational benefits from this student-centered approach.

## Supplementary Information

Supplementary Material 1.

Supplementary Material 2: Supplementary table: Correlation coefficient between factors CBL .

## Data Availability

Some data are not publicly available due to ethical and privacy restrictions. Other datasets used and/or analyzed during this study are available from the corresponding author upon reasonable request.

## Abbreviations

CBL: Case-based learning
IRB: Institutional Review Board
KSU: King Saud University
PBL: Problem based learning
FDP: Faculty development program
IBM: International Business Machines
SPSS: Statistical Package for the Social Sciences
