# Supplementary material for: Medical students’ perceptions of the impact of case-based learning on engagement, cognitive skills, and communication
Source: BMC Med Educ. 2026 Apr 28;26:958. doi: 10.1186/s12909-026-09316-2 (PMC13261970; doi:10.1186/s12909-026-09316-2)
Supplement: Supplementary file 1 — Supplementary Material 1. [file 12909_2026_9316_MOESM1_ESM.pdf]

Questionnaire of CBL

1. Gender
2. Current Academic Year:
3. In your medical curriculum, how often do you have the opportunity to engage in case-based learning?
4. Rate your overall satisfaction with the case-based learning sessions:
5. To what extent do you believe case-based learning enhances your understanding of medical concepts?
6. Using case-based learning is beneficial for understanding deep learning and developing critical thinking skills
7. A CBL provides the platform for developing and understanding the sound knowledge of a core subject.
8. Do case-based learning skills help you prepare for a future career in medicine?
9. Have case-based learning sessions improved your problem-solving skills?
10. Do you think case-based learning has contributed to better retention of medical knowledge compared to traditional teaching methods?
11. Did the discussion during the CBL session improve teamwork skills?
12. Is clinical reasoning improved by CBL sessions?
13. To what extent do case-based learning activities encourage collaboration with your peers?
14. How has case-based learning contributed to the development of your communication skills in a medical context?
15. To what extent do you think technology (e.g., virtual cases, simulations) enhances the effectiveness of case-based learning in clinical teaching and clerkship training?
16. Do you feel that case-based learning helps bridge the gap between theoretical knowledge and its practical application in real-world clinical scenarios?
17. In your experience, what challenges, if any, have you encountered in understanding and adapting to the case-based learning format?
18. Were there any difficulties in accessing or utilizing the necessary resources for case-based learning?
19. Have there been challenges in maintaining student engagement and motivation throughout case-based learning sessions?
