# Supplementary material for: Medical students’ perceptions of the impact of case-based learning on engagement, cognitive skills, and communication
Source: BMC Med Educ. 2026 Apr 28;26:958. doi: 10.1186/s12909-026-09316-2 (PMC13261970; doi:10.1186/s12909-026-09316-2)
Supplement: Supplementary file 2 — Supplementary Material 2: Supplementary table: Correlation coefficient between factors CBL . [file 12909_2026_9316_MOESM2_ESM.docx]

**Supplementary table: Correlation coefficient between factors CBL**

| **Question** | **1** | **2** | **3** | **4** | **5** | **6** | **7** | **8** | **9** | **10** | **11** |
| --- | --- | --- | --- | --- | --- | --- | --- | --- | --- | --- | --- |
| 1. I am satisfied with the case-based learning sessions. | 1 | 0.60  (<0.001) | 0.47  (<0.001) | 0.48  (<0.001) | 0.37  (<0.001) | 0.44  (<0.001) | 0.43  (<0.001) | 0.41  (<0.001) | 0.49  (<0.001) | 0.30  (<0.001) | 0.48  (<0.001) |
| 2. I believe that case-based learning enhances my understanding of medical concepts. | 0.60 (<0.001) | 1 | 0.71  (<0.001) | 0.67  (<0.001) | 0.58  (<0.001) | 0.64  (<0.001) | 0.65  (<0.001) | 0.35  (<0.001) | 0.61  (<0.001) | 0.31  (<0.001) | 0.64  (<0.001) |
| 3. Using case-based learning is beneficial for deep learning and developing critical thinking skills. | 0.47  (<0.001) | 0.71  (<0.001) | 1 | 0.74  (<0.001) | 0.64  (<0.001) | 0.60  (<0.001) | 0.70  (<0.001) | 0.43  (<0.001) | 0.68  (<0.001) | 0.30  (<0.001) | 0.78  (<0.001) |
| 4. The CBL session provides the platform for developing and understanding the sound knowledge of a core subject. | 0.48  (<0.001) | 0.67  (<0.001) | 0.74  (<0.001) | 1 | 0.62  (<0.001) | 0.55  (<0.001) | 0.67  (<0.001) | 0.54  (<0.001) | 0.68  (<0.001) | 0.34  (<0.001) | 0.75  (<0.001) |
| 5. I believe that the skills acquired from case-based learning sessions would help me prepare for a future career in medicine. | 0.37  (<0.001) | 0.58  (<0.001) | 0.64  (<0.001) | 0.62  (<0.001) | 1 | 0.58  (<0.001) | 0.62  (<0.001) | 0.37  (<0.001) | 0.62  (<0.001) | 0.31  (<0.001) | 0.63  (<0.001) |
| 6. Case-based learning sessions improved my problem-solving skills. | 0.44  (<0.001) | 0.64  (<0.001) | 0.60  (<0.001) | 0.55  (<0.001) | 0.58  (<0.001) | 1 | 0.65  (<0.001) | 0.39  (<0.001) | 0.53  (<0.001) | 0.33  (<0.001) | 0.53  (<0.001) |
| 7. Case-based learning has contributed to better retention of medical knowledge compared to traditional teaching methods. | 0.43  (<0.001) | 0.65  (<0.001) | 0.70  (<0.001) | 0.67  (<0.001) | 0.62  (<0.001) | 0.65  (<0.001) | 1 | 0.48  (<0.001) | 0.66  (<0.001) | 0.26  (<0.001) | 0.67  (<0.001) |
| 8. The discussion during the CBL sessions improved my teamwork skills. | 0.41  (<0.001) | 0.35  (<0.001) | 0.43  (<0.001) | 0.54  (<0.001) | 0.37  (<0.001) | 0.39  (<0.001) | 0.48  (<0.001) | 1 | 0.43  (<0.001) | 0.28  (<0.001) | 0.49  (<0.001) |
| 9. I believe my clinical reasoning has improved with CBL sessions. | 0.49  (<0.001) | 0.61  (<0.001) | 0.68  (<0.001) | 0.68  (<0.001) | 0.62  (<0.001) | 0.53  (<0.001) | 0.66  (<0.001) | 0.43  (<0.001) | 1 | 0.27  (<0.001) | 0.68  (<0.001) |
| 10. The adoption of technology (e.g., virtual cases, and simulations) enhances the effectiveness of case-based learning in clinical teaching and clerkship training. | 0.30  (<0.001) | 0.31  (<0.001) | 0.30  (<0.001) | 0.34  (<0.001) | 0.31  (<0.001) | 0.33  (<0.001) | 0.26  (<0.001) | 0.28  (<0.001) | 0.27  (<0.001) | 1 | 0.39  (<0.001) |
| 11. I feel that case-based learning helps bridge the gap between theoretical knowledge and its practical application in real-world clinical scenarios. | 0.48  (<0.001) | 0.64  (<0.001) | 0.78  (<0.001) | 0.75  (<0.001) | 0.63  (<0.001) | 0.53  (<0.001) | 0.67  (<0.001) | 0.49  (<0.001) | 0.68  (<0.001) | 0.39  (<0.001) | 1 |
